# Zygotic activin A is dispensable for the mouse preimplantation embryo development and for the derivation and pluripotency of embryonic stem cells^†^

**DOI:** 10.1093/biolre/ioae156

**Published:** 2024-11-06

**Authors:** Eliza Winek, Lidia Wolińska-Nizioł, Katarzyna Szczepańska, Anna Szpakowska, Olga Gewartowska, Izabela Wysocka, Magdalena Grzesiak, Aneta Suwińska

**Affiliations:** Department of Embryology, Institute of Developmental Biology and Biomedical Sciences, Faculty of Biology, University of Warsaw, Warsaw, Poland; Department of Embryology, Institute of Developmental Biology and Biomedical Sciences, Faculty of Biology, University of Warsaw, Warsaw, Poland; Department of Embryology, Institute of Developmental Biology and Biomedical Sciences, Faculty of Biology, University of Warsaw, Warsaw, Poland; Department of Embryology, Institute of Developmental Biology and Biomedical Sciences, Faculty of Biology, University of Warsaw, Warsaw, Poland; Laboratory of RNA Biology, International Institute of Molecular and Cell Biology, Warsaw, Poland; Department of Embryology, Institute of Developmental Biology and Biomedical Sciences, Faculty of Biology, University of Warsaw, Warsaw, Poland; Department of Embryology, Institute of Developmental Biology and Biomedical Sciences, Faculty of Biology, University of Warsaw, Warsaw, Poland; Department of Embryology, Institute of Developmental Biology and Biomedical Sciences, Faculty of Biology, University of Warsaw, Warsaw, Poland

**Keywords:** activin A, *Inhba*, preimplantation embryo, blastocyst, embryonic stem cells

## Abstract

In this work, we aimed to determine the role of activin A during crucial events of mouse embryogenesis and distinguish the function of the protein of zygotic origin and the one secreted by the maternal reproductive tract. To this end, we recorded the progression of development and phenotype of *Inhba* knockout embryos and compared them with the heterozygotes and wild-type embryos using time-lapse imaging and detection of lineage-specific markers. We revealed that the zygotic activin A deficiency does not impair the course and rate of development of embryos to the blastocyst stage. *Inhba* knockout embryos form functional epiblast, as evidenced by their ability to give rise to embryonic stem cells. Our study is the first to show that derivation, maintenance in culture, and pluripotency of embryo-derived embryonic stem cells are exogenous and endogenous activin A independent. However, the implantation competence of activin A–deficient embryos may be compromised as indicated in the outgrowth assay.

## Teaser

**Summary Sentence**

The knockout of zygotic activin A affects neither the development of embryos to the blastocyst stage nor the derivation and maintenance of embryonic stem cells; however, it may impair the ability of embryos to implantation.

## Graphical Abstract

**Graphical Abstract:**
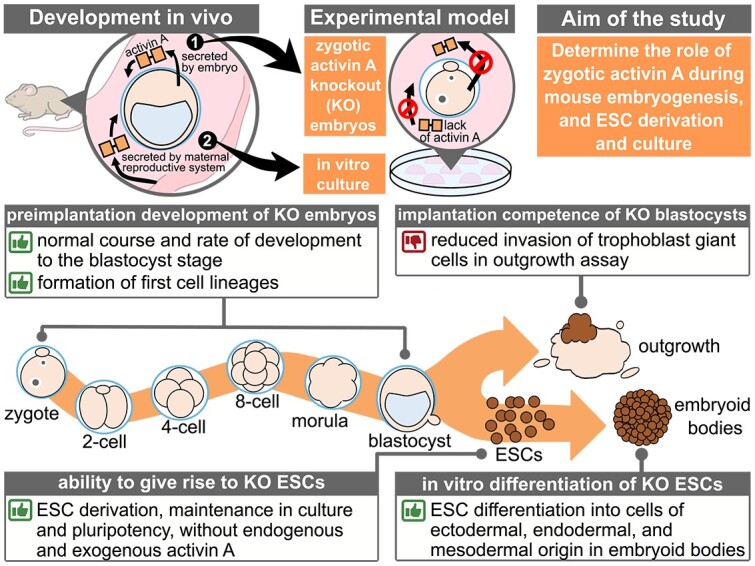

## Introduction

The successful development of mammalian, including mouse and human, embryos is determined by growth factors secreted by both the mother’s reproductive system and the embryo itself. Encoded by the *Inhba* gene activin A, a member of the transforming growth factor beta (TGFβ) family, is an example of such a multiple-source protein. Although dysregulation of the activin A signaling pathway in humans is correlated with the occurrence of miscarriages and ectopic pregnancies [1], due to the various origins of this protein, its exact role in mammalian embryonic development remains ambiguous.

A stock of maternal activin A is present in the mouse oocyte, undergoing gradual degradation during early cleavage stages [2]. At the 2- and 4-cell stages, the expression of activin A decreases, and increases again by the 8-cell stage, reaching a maximum at the compacted morula probably due to translation of zygotic mRNA. At the E3.5 blastocyst stage, this protein is confined to the inner cell mass (ICM), a future embryo body. At the peri-implantation E4.5 blastocyst, activin A is present in the trophectoderm (TE), which is responsible for the formation of the embryonic part of the placenta and implantation of the embryo in the uterus [2–4]. Moreover, activin A is also present in human embryos throughout preimplantation development. Although the level of activin A in humans is very low up to the morula stage, it increases significantly at the blastocyst stage [5].

It was also shown that in the mouse and human, activin A is produced and secreted by the ovary, oviduct, and epithelium of the uterus [2–4]. Activin receptors are present in the oocyte, early cleavage stage embryo, blastocyst, trophoblast, and decidua [5–8], making them responsive to growth factors secreted by the reproductive tract and allowing for crosstalk mediated by activin during fertilization, preimplantation, and implantation events.

In the early stages of mouse postimplantation development (E6.0–8.5), activin A is not expressed, but high levels of this protein have been observed in the maternal decidua [9–11]. It has been suggested, but never directly proven, that exogenous activin A produced by the uterus acts in a paracrine manner, inducing mesoderm formation and gastrulation in the developing embryo [11]. Interestingly, although activin A is present in the preimplantation stages of mouse embryonic development, the lack of this protein does not preclude successful development to term. Activin A–deficient mice are born but die only within 24 h of birth because of craniofacial defects [12]. However, the lack of an early phenotype in loss-of-function mutants of components of the activin signaling pathway [13] may be masked by the presence of exogenous activin A (derived from a female’s reproductive tract).

Consistent with the observation that activin A is expressed in the ICM of E3.5 blastocysts, high mRNA levels for activin A are also present in mouse and human embryonic stem cells (ESCs) [3, 14, 15]. It is known that human ESCs (hESCs), unlike mouse ESCs (mESCs), require the presence of exogenous activin A to maintain their undifferentiated nature and pluripotency [15–17]. However, the function of endogenous activin A in both murine and human embryonic stem cells has remained unknown so far.

In this work, we investigated the consequences of loss of zygotic activin A to determine the requirement for this protein in the specification of cell lineages in the mouse embryo and also for the isolation and maintenance of ESC lines. To observe developing embryos continuously and individually, we recorded their development in vitro from the zygote (to exclude the impact of activin A from the reproductive tract) to the blastocyst stage using time-lapse imaging. Our data suggest that the zygotic pool of activin A is not necessary for correct preimplantation embryo development. Activin A–deficient embryos specify primary cell lineages properly, reaching the blastocyst stage, however, with the limited invasion of trophoblast cells during the implantation process as suggested by the blastocyst outgrowth assay. Our data also show, for the first time, that endogenous and exogenous activin A is not required for the derivation of mouse ESC lines and maintaining their pluripotency. The absence of functional activin A does not prevent the in vitro differentiation of ESCs into three germ layers, suggesting redundancy of this protein for the initial stages of gastrulation during mouse postimplantation development.

## Results

### Knockout of the first exon of the activin A gene in the mouse results in a characteristic phenotype and early lethality of the newborns

**Figure 1:**
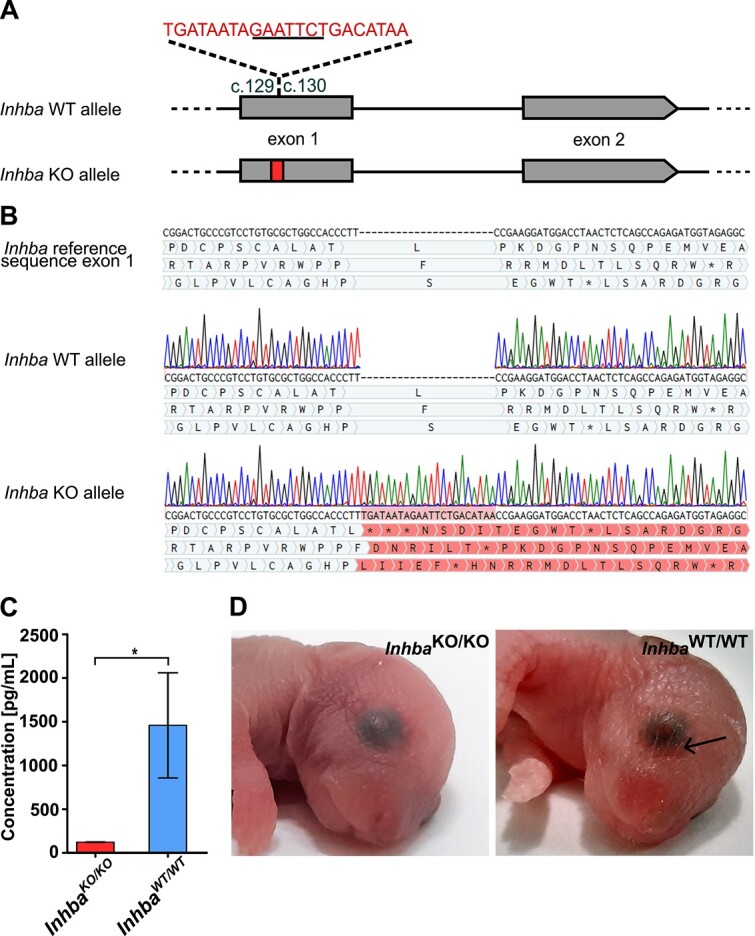
Knockout of the first exon of activin A gene results in a characteristic phenotype of the newborn mice. (A) Scheme of the *Inhba*-KO mutation involving cassette insertion between 29th and 30th nucleotides of exon 1 of the *Inhba* gene. The cassette contains the STOP codon and EcoRI enzyme digestion sequences (underlined). (B) Sanger sequencing data showing insertion of 22-bp cassette (nucleotides sentence listed above exon 1) in coding exon 1 *Inhba* in the *Inhba*-KO allele, compared to the reference sequence from the Ensembl database and sequencing results for WT allele. The insertion causes premature translation arrest in each of the three possible open reading frame (ORF) sequences (^*^STOP codon), and results in frameshift mutation (highlighted amino acids). (C) Level of activin A protein in the culture medium of mouse embryonic fibroblasts (MEFs). The results of ELISA analyses show that MEFs derived from *Inhba*^KO/KO^ mice (*n* = 3) have above 11-fold reduced concentration of activin A relative to *Inhba*^WT/WT^ MEFs derived from *Inhba*^WT/WT^ siblings (*n* = 3; *P* < 0.05, two-tailed Student *t*-test). (D) Phenotype of *Inhba* mutant mice. There is a visible absence of whiskers in knockout *Inhba*^KO/KO^ mice, compared to wild-type *Inhba*^WT/WT^ mice.

To investigate the role of endogenous activin A in preimplantation mouse embryo development, we used the CRISPR/Cas9 method to obtain a mouse line with a knockout of the zygotic activin A gene (*Inhba-*KO). The *Inhba*-KO line was established by a loss-of-function indel mutation in coding exon 1 of the *Inhba* gene in C57BL/6/Tar x CBA/Tar mixed background and C57BL/6/Tar inbred background. The insert cassette contains STOP codon sequences, which cause premature translation arrest in each of the three possible open reading frame (ORF) sequences and result in frameshift mutation (Figure 1A), which we confirmed by Sanger sequencing (Figure 1B). Furthermore, the lack of activin A protein in knockout (*Inhba*^KO/KO^) mouse was endorsed in the enzyme-linked immunosorbent assay (ELISA) from the culture medium of mouse embryonic fibroblasts (MEFs) derived from *Inhba*^KO/KO^ and wild-type (*Inhba*^WT/WT^) fetuses (Figure 1C).

To verify the phenotype of *Inhba-*KO mice, we intercrossed heterozygous (*Inhba*^KO/WT^) females with males of the same genotype. Since *Inhba*^KO/KO^ mutation is lethal soon after birth, we recovered the fetuses at E19 and correlated their phenotype with the genotype. In the case of the *Inhba-*KO mouse line, we overall obtained 15 fetuses, 4 of which had the *Inhba*^KO/KO^ genotype. *Inhba*^KO/KO^ homozygotes from both mouse lines displayed a characteristic phenotype described by Matzuk et al. [12]: lack of whiskers and lower incisors (Figure 1D).

Nevertheless, the lack of an early mutant phenotype in zygotic knockout embryos may be masked by the presence of exogenous activin A derived from a female’s reproductive tract, as it was proved that exogenous protein facilitates embryonic development in vitro [18–22]*.*

### Lack of activin A does not impair the course and rate of preimplantation mouse embryo development

Based on the premise that insight into the role of endogenous activin A is only possible in the absence of an exogenous protein, we cultured knockout (*Inhba*^KO/KO^), heterozygotes (*Inhba*^KO/WT^), and wild-type (*Inhba*^WT/WT^) embryos in vitro from the zygote to the blastocyst stage. We used embryos in two genetic backgrounds (C57BL/6/Tar x CBA/Tar mixed background and on an inbred C57BL/6 genetic background). To compare the phenotype of *Inhba*^KO/KO^ embryos with stage-matched *Inhba*^KO/WT^ and *Inhba*^WT/WT^ embryos, we analyzed the morphokinetic parameters by recording the progress of their development using time-lapse microscopy.

Based on these observations, we determined no difference in the development efficiency to the blastocyst stage between embryos of different genotypes. Out of 33 *Inhba*^KO/KO^ embryos in C57BL/6/Tar x CBA/Tar genetic background subjected to time-lapse observations, 31 (94%) developed to the blastocyst and 2 (6%) failed to reach the blastocyst stage (arrested embryos). These percentages were comparable with the data for *Inhba*^KO/WT^ and *Inhba*^WT/WT^ control embryos [102/111 (92%) and 30/37 (81%) of blastocysts, respectively] (Chi-square test; *P* > 0.05).

**Figure 2:**
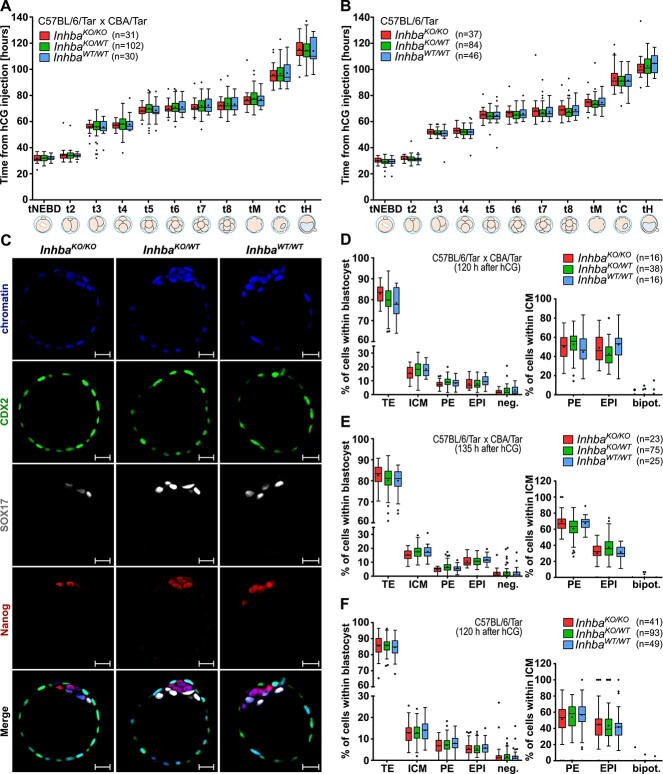
Embryos deprived of zygotic and exogenous activin A undergo normal development to blastocyst stage. (A, B) Comparison of main morphokinetic parameters between *Inhba*^KO/KO^ and control—*Inhba*^KO/WT^ and *Inhba*^WT/WT^ embryos in C57BL/6/Tar x CBA/Tar (A) and C57BL/6/Tar (B) genetic background. The value of individual parameters was calculated as the number of hours from the injection of hCG to the time of: t_NEBD_—breakdown of nuclear envelopes of pronuclei; t_2_–t_8_—complete division to the certain number of blastomeres; tM—compaction; tC and tH—beginning of cavitation and hatching process, respectively. On all graphs, the total number of embryos analyzed for each genotype is provided in parentheses. On the boxplots, the middle lines represent medians, the cross shows the mean value, the hinges indicate the interquartile range, the whiskers represent the minimum and maximum values in the group, and the dots show outliers. The lack of statistically significant (*P* > 0.05) changes was proved by the Kruskal–Wallis test separately for each morphokinetic parameter. (C) Confocal images of blastocysts in C57BL/6/Tar x CBA/Tar genetic background immunostained with antibodies against CDX2 (TE), SOX17 (PE), Nanog (EPI), and chromatin stained with Chromomycin A3. Scale bars: 20 μm. (D–F) Percentage contribution of primary cell lineages, negative and bipotential cells within blastocyst and inner cell mass in: 120 h C57BL/6/Tar x CBA/Tar blastocysts (D), 135 h C57BL/6/Tar x CBA/Tar blastocysts (E), and 120 h C57BL/6/Tar blastocysts (F) (*P* > 0.05, Kruskal–Wallis test). Abbreviations: trophectoderm (TE), inner cell mass (ICM), primitive endoderm (PE), epiblast (EPI), negative (neg.; CDX2−SOX17−Nanog−) cells, bipotential (bipot.; Nanog+SOX17+) cells. See also Figure S1, and Movie 1 and Movie 2.

The data for the morphokinetic parameters analyzed for embryos that developed to the blastocyst stage are presented in Figure 2A and Figure S1A. Based on our measurements, we did not notice a significant difference in the dynamics of development between *Inhba*^KO/KO^ and both groups of control embryos (*Inhba*^KO/WT^ and *Inhba*^WT/WT^). The timing of the nuclear envelope breakdown of pronuclei (t_NEBD_), cleavage divisions (t_2_–t_8_), durations of cell cycles (m_1_, cc_2_, and cc_3_), and synchrony of the cleavage rounds (s_2_ and s_3_) were comparable in both knockout and control embryos (Figure 2A; Figure S1A; Movie 1; Kruskal–Wallis test; *P* > 0.05). No differences in compaction (tM) and cavitation (tC) rates were also observed between *Inhba*^KO/KO^ embryos and both controls (Figure 2A; Movie 1; Kruskal–Wallis test; *P* > 0.05). Since subtle phenotypes might be missed in knockout embryos generated in the mixed background, we repeated the same analyses using knockout mice in the inbred C57BL/6 background and received comparable results (Figure 2B; Figure S1B, Movie 2).

### Activin A knockout does not affect the formation of the first cell lineages of the blastocyst

In order to determine the effect of depletion of zygotic *activin A* expression on cell lineage specification, we detected the presence of Nanog/SOX2, SOX17/GATA4, and CDX2, markers of EPI, PE, and TE, respectively (Figure 2C and Figure S1C). To observe the process of sorting the PE and EPI within the blastocyst, we captured the embryos at two time points: 120 h and 135 h after hCG injection. Immunofluorescent analysis revealed that neither 120 h (*n* = 16) nor 135 h (*n* = 23) *Inhba*^KO/KO^ embryos significantly differed in the number and percentage of cells contributing to the cell lineages (EPI, PE, and TE) from blastocysts of other genotypes [*Inhba*^KO/WT^: 120 h (*n* = 38), 135 h (*n* = 75), and *Inhba*^WT/WT^: 120 h (*n* = 16), 135 h (*n* = 25); Figure 2D and E, respectively]. We performed a similar analysis for embryos in the inbred C57BL/6 background and also did not observe any differences between the embryos of various genotypes (Figures 2F and S1C).

Furthermore, to exclude the possibility that the penetrance of a mutation may be contingent upon the environmental stress (as previously described in [23]), we cultured *Inhba*^KO/KO^, *Inhba*^KO/WT^, and *Inhba*^WT/WT^ embryos from the zygote to the blastocyst stage in conditions more closely resembling those prevailing in vivo*.* We reduced the O_2_ level to 5% and replaced the M16 medium with a more abundant KSOM medium, additionally modified by increasing the concentration of glucose and essential amino acids (EAAs). However, despite these modifications in culture conditions, *Inhba*^KO/KO^ embryos reached the blastocysts stage and had properly formed TE, EPI, and PE cell lineages (Figure S2). Overall, our data indicate that activin A is not required for the correct development of the embryo to the blastocyst stage and specification of the first cell lineages.

### Activin A deficiency impairs the implantation competence of blastocysts

The expression of activin A in the TE of the E4.5 blastocyst [9] suggests a role of this protein in implantation. Since we showed that *Inhba*^KO/KO^ embryos are able to develop until the blastocyst stage, we next investigated whether they have functional trophectoderm capable of implantation.

We could not apply uterine embryo transfer to assess the functionality of the TE because activin A is also secreted by maternal tissues [3, 4, 6, 24, 25]. Therefore, we verified the ability of activin A–deficient blastocysts to the formation of outgrowths on gelatin-coated plates, which constitutes the reliable and widely used in vitro model of embryo attachment and the initial stages of implantation [26, 27].

**Figure 3:**
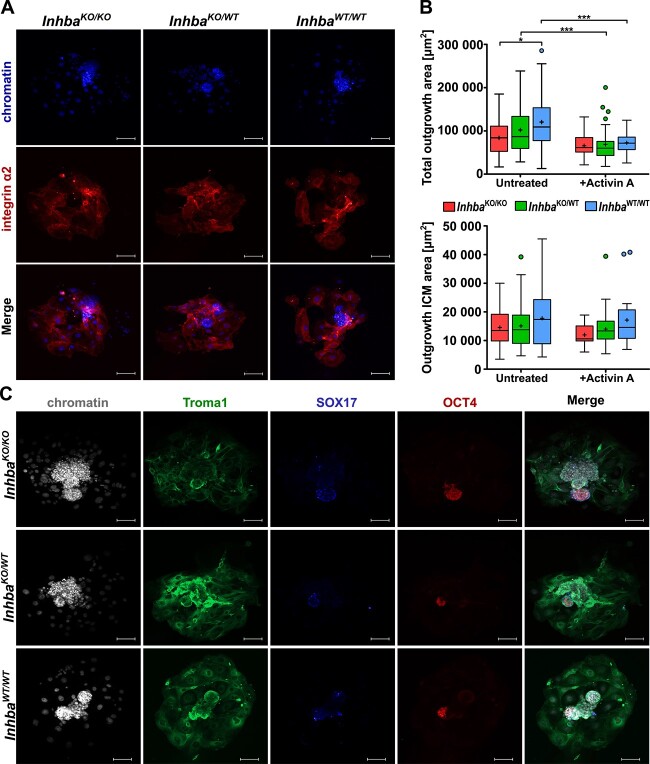
Activin A deficiency affects outgrowth formation. (A) Confocal images of outgrowths immunostained with antibodies against adhesion protein integrin α2, with chromatin stained with Hoechst 33342. (B) The total surface area and the inner cell mass (ICM) area of *Inhba*^KO/KO^ (*n* = 22), *Inhba*^KO/WT^ (*n* = 54), and *Inhba*^WT/WT^ (*n* = 45) untreated outgrowths cultured in standard medium and *Inhba*^KO/KO^ (*n* = 22), *Inhba*^KO/WT^ (*n* = 45), and *Inhba*^WT/WT^ (*n* = 21) outgrowths cultured with recombinant activin A. On the boxplots, the middle lines represent medians, the cross shows the mean value, the hinges indicate the interquartile range, the whiskers represent the minimum and maximum values in the group, and the dots show outliers. The total surface area and ICM area between *Inhba*^KO/KO^, *Inhba*^KO/WT^, and *Inhba*^WT/WT^ outgrowths were analyzed by Kruskal–Wallis test with post hoc Dunn test, while untreated and cultured with activin A outgrowths of the same genotype were compared with Mann–Whitney *U*-test: ^*^P < 0.05; ^***^P < 0.001. (C) Confocal images of outgrowths immunostained with antibodies against trophoblast giant cell marker Troma1, a marker of primitive endoderm—SOX17, and pluripotency marker OCT4, with chromatin stained with Hoechst 33342. Scale bar in all confocal images: 50 μm.

We revealed that although *Inhba*^KO/KO^ blastocysts formed outgrowths at a similar efficiency to the corresponding control embryos (Table 1; *P* > 0.05, Chi-square test), the observed genotype distribution of obtained outgrowths was inconsistent with the expected Mendelian ratios, as activin A–deficient outgrowths were less frequent than those of other genotypes (Table 1; *P* < 0.01, Chi-square goodness-of-fit test). Since we were able to genotype only those outgrowths that remain attached to the end of the culture, we decided to examine whether activin A deficiency affects the adhesion abilities of the TE. We immunostained the obtained outgrowths for the presence of integrin α2, one of the proteins engaged in cell adhesion. However, we did not notice the difference in the level of this protein between activin A–lacking outgrowths and controls (Figure 3A).

**Table 1:** *Inhba* ^KO/KO^ blastocysts formed outgrowths with comparable efficiency to *Inhba*^KO/WT^ and *Inhba*^WT/WT^ embryos, although activin A–deficient outgrowths are less frequent than other genotypes

**Genotype**	**No. of blastocysts put into culture**	**Obtained outgrowths [no. (%)]** ^**a**^	**Observed genotype distribution of outgrowths [%]** ^**b**^	**Expected genotype distribution [no. (%)]** ^**b**^	**Outgrowths without ICM [no. (%)]** ^**a**^
***Inhba*** ^***KO/KO***^	22	22 (100%)	18%^**^	30 (25%)	0 (0%)
***Inhba*** ^***KO/WT***^	62	54 (87%)	45%^**^	61 (50%)	7 (13%)
***Inhba*** ^***WT/WT***^	48	45 (94%)	37%^**^	30 (25%)	1 (2%)
**Total (%)**		121 (100%)	100%	121 (100%)	

^a^ *P* > 0.05, Chi-square test

^b^In accordance with Mendel’s First Law, 1:2:1 (25%:50%:25%)

^^**^^ *P* < 0.01, Chi-square goodness-of-fit test between obtained and expected genotype distribution of outgrowths

no.—number

Next, the rate of trophoblast cell migration was determined by measuring the mean area of outgrowing embryos (the only ICM outgrowth and total area including trophoblast giant cells) using an image analysis system. Importantly, although the area of ICM outgrowths was comparable between different genotypes (*P* > 0.05, Kruskal–Wallis test), activin A–deficient outgrowths displayed a smaller spreading area of TE cells than wild-type control outgrowths (84,016 μm^2^ vs. 1,090,070 μm^2^, respectively; *P* < 0.05, Kruskal–Wallis test with post hoc Dunn test) (Figure 3B). Despite the observed difference, the obtained *Inhba*^KO/KO^ outgrowths contained all cell lineages, as shown by the presence of their markers: OCT4 (EPI), SOX17 (PE), and Troma1 (trophoblast giant cells) (Figure 3C). Then, we attempted to rescue the *Inhba*^KO/KO^ outgrowths phenotype by supplementing the culture medium with recombinant activin A. We revealed that treatment of outgrowths with activin A abolished the difference in the spreading area of TE between *Inhba*^KO/KO^ and *Inhba*^WT/WT^ outgrowths (Figure 3B). However, unexpectedly, adding exogenous activin A negatively affected the size of *Inhba*^KO/WT^ and *Inhba*^WT/WT^ outgrowths. Their spreading area was smaller in comparison to the corresponding untreated outgrowths (*P* < 0.001; Mann–Whitney *U*-test; Figure 3B). Of note, the area of the ICM in outgrowths of all genotypes was similar with or without exogenous activin A (*P* > 0.05; Mann–Whitney *U*-test) and the ICM area of the *Inhba*^KO/KO^ outgrowths was not significantly different from the control outgrowths when the culture was supplemented with activin A (*P* > 0.05; Kruskal–Wallis test; Figure 3B).

Taken together, the outgrowth formation assay showed reduced invasion of trophoblast cells in *Inhba*^KO/KO^ outgrowths, which was abolished when the outgrowths were cultured in the presence of exogenous activin A. However, in control groups, a high dose of activin A uniformly impaired trophoblast invasion as evidenced by a decrease in the total surface area of blastocyst outgrowths compared to the corresponding untreated variants.

### Activin A–deficient blastocysts can be a source of pluripotent ESCs

Activin A is present in the ICM of the mouse blastocyst and ESCs [3, 14]. In contrast to human ESCs, this protein is not routinely added during mouse ESC derivation from preimplantation embryos. However, it is unclear whether endogenous activin A is required for ESC derivation. Thus, we decided to determine whether we could derive ESCs from *Inhba*^KO/KO^ embryos.

Out of 25 embryos obtained from *Inhba*^KO/WT^ intercrosses, we established 11 ESC lines. Of all ESC lines isolated, 3 (27%) were *Inhba*^WT/WT^, 5 were *Inhba*^KO/WT^ (46%), and 3 were *Inhba*^KO/KO^ (27%) (Figure S3A; *P* > 0.05, Chi-square goodness-of-fit test). Colonies of ESCs that lack functional activin A (*Inhba*^KO/KO^) did not differ morphologically from the corresponding ESC colonies of other genotypes (*Inhb*^WT/WT^ and *Inhba*^KO/WT^) (Figure S3B). Next, ESCs were cultured under conditions that supported pluripotency and self-renewal, i.e. in a medium containing LIF, and then were processed for immunolocalization of pluripotency and differentiation markers. We showed that activin A–deficient ESCs, similarly to *Inhba*^WT/WT^ and *Inhba*^KO/WT^ ESCs, expressed markers specific for the epiblast (SOX2, Nanog) (Figure S3C and D, respectively). Moreover, we did not notice the presence of CDX2 and SOX17, markers of TE and PE, respectively, regardless of the genotype (Figure S3E and F).

**Figure 4:**
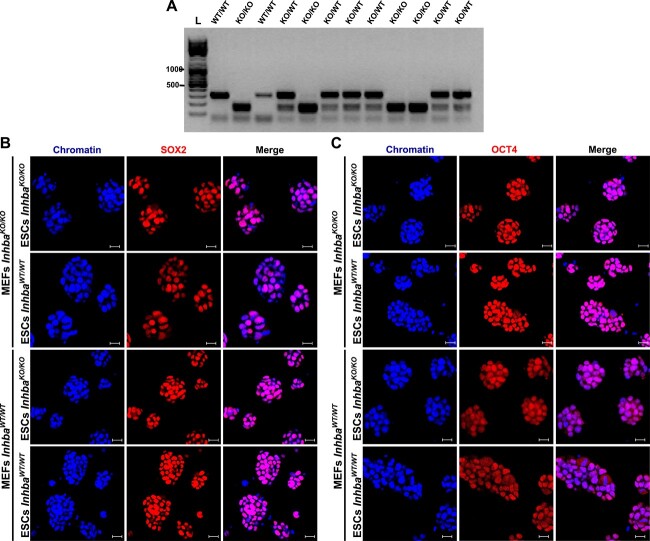
Mouse ESCs can be established in the absence of endogenous activin A and exogenous protein provided by the MEF feeder layer. (A) Gel image for genotyping-derived *Inhba*^KO/KO^, *Inhba*^KO/WT^, and *Inhba*^WT/WT^ ESCs of *Inhba*-KO line upon the feeder layer of *Inhba*^KO/KO^ MEFs. The wild-type allele results in a 277-bp product, while the *Inhba*-KO allele results in a 299-bp product and gives two bands of 171 and 128 bp after digestion with EcoRI. (B, C) Confocal images of *Inhba*^KO/KO^ and *Inhba*^WT/WT^ ESCs cultured upon the feeder layer of *Inhba*^KO/KO^ or *Inhba*^WT/WT^ MEFs. All ESCs were immunostained with antibodies against pluripotency markers: SOX2 (B) and OCT4 (C), with chromatin stained with Chromomycin A3. Scale bar: 20 μm. See also Figure S4.

As we have shown that wild-type MEFs are also a source of activin A (Figure 1C), it is important to exclude the possibility that the derivation of ESCs from *Inhba*^KO/KO^ embryos is only possible provided that an exogenous source of this protein is present. Thus, we decided to determine whether we could derive ESCs from *Inhba*^KO/KO^ embryos upon the feeder layer of *Inhba*^KO/KO^ MEFs. Out of 16 embryos, we managed to establish 12 ESC lines. Of the ESC lines isolated, four (33%) were *Inhba*^KO/KO^ (Figure 4A), indicating that ESCs can be established in the absence of endogenous activin A regardless of the exogenous protein provided by the feeder layer. Moreover, knockout ESCs were indistinguishable from wild-type ESCs in terms of morphology and expression of pluripotency (Figures 4B and C, and S4A) and differentiation markers (Figure S4B and C) regardless of whether they were exposed to an external source of activin A provided by the feeder layer.

The above results indicate that *Inhba*^KO/KO^ embryos can give rise to ESCs with the expected frequency. Moreover, ESCs can be isolated and maintained in culture in the undifferentiated state in the absence of endogenous and exogenous activin A.

### Activin A–lacking ESCs are able to differentiate into three germ layers

**Figure 5:**
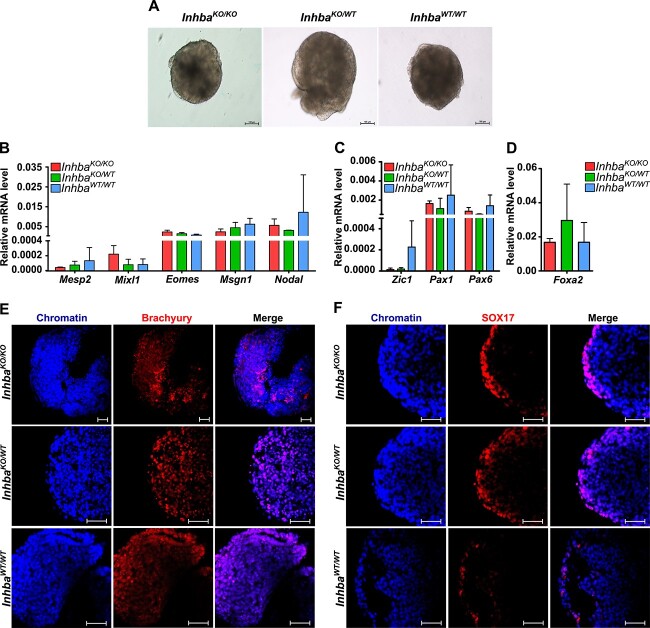
Zygotic activin A is dispensable for the differentiation of ESCs into three germ layers. (A) Bright-field images of *Inhba*^KO/KO^, *Inhba*^KO/WT^, and *Inhba*^WT/WT^ embryoid bodies. (B–D) Relative expression level of mesoderm marker genes (B), ectoderm including neuroectoderm marker genes (C), and endoderm gene (D). The bars represent mean values from three biological replicates of 50 embryoid bodies used for each sample, error bars represent SD; *P* > 0.05 reported for comparison in one-way ANOVA test for each gene. (E, F) Confocal images of embryoid bodies immunostained with antibodies against mesoderm marker—Brachyury (E) and endoderm marker—SOX17 (F), with chromatin stained with Chromomycin A3. Scale bar: 50 μm.

Next, we compared the ability of *Inhba*^KO/KO^ and control (heterozygous and wild-type) ESCs to differentiate into tissues originating from three germ layers: ectoderm, mesoderm, and endoderm. To this end, we tested whether they could differentiate in vitro in embryoid bodies (EBs). We showed that all ESC lines analyzed could form embryoid bodies regardless of the genotype. No differences, neither in morphology nor size, were observed between knockout and control EBs (Figure 5A). Similarly to control embryoid bodies, *Inhba*^KO/KO^ EBs expressed the mesodermal markers: Mesogenin 1 (*Msgn1*), Mesoderm posterior BHLH transcription factor 2 (*Mesp2*), Nodal, Eomesodermin (*Eomes*), Mix Paired-like Homeobox (*Mixl1*) (Figure 5B), the ectodermal markers: Zic Family Member 1 (*Zic1*), Paired Box 1 (*Pax1*), and Paired Box 6 (*Pax6*) (Figure 5C), and the endodermal marker Forkhead Box A2 (*Foxa2*) (Figure 5D) as indicated by RT-qPCR analyses.

Among the mesodermal markers, the highest relative expression level was recorded for *Nodal* and the lowest for *Mesp2* associated with primitive streak formation (Figure 5B). The averaged expression levels of all analyzed mesodermal markers (*Mesp2*, *Mixl1*, *Eomes*, *Msgn1,* and *Nodal*) were at a similar level in EBs lacking activin A to both control genotypes (*Inhba*^WT/WT^ and *Inhba*^KO/WT^) (Figure 5B; *P* > 0.05, one-way analysis of variance (ANOVA) for each).

The average results showing mRNA expression for ectoderm (*Zic1*, *Pax1*) and neuroectoderm (*Pax6*) markers are presented in Figure 5C. The highest expression level was observed for *Pax1* and the lowest for *Zic1*. The levels of mRNA transcripts that encoded *Zic1*, *Pax1*, and *Pax6* were similar in all EBs regardless of their genotype (Figure 5C; *P* > 0.05, one-way ANOVA for each).

The results of the average expression of the endoderm marker *Foxa2* showed that *Inhba*^KO/KO^ EBs were characterized by comparable expression of this gene compared to *Inhba*^KO/WT^ and wild-type EBs (Figure 5D; *P* > 0.05, one-way ANOVA test).

The ability of *Inhba*^KO/KO^ to multidirectional differentiation shown by qPCR analysis was confirmed by showing the expression of endodermal and mesodermal markers at the protein level by immunolocalization. All EBs tested, regardless of the genotype, contained cells synthesizing Brachyury and SOX17, which are specific for mesoderm and endoderm, respectively (Figure 5E and F). Thus, the presented results clearly demonstrate that *Inhba*^KO/KO^ ESCs are pluripotent, as they can differentiate in vitro into cells of ectodermal, endodermal, and mesodermal origin.

## Discussion

Activin A is a potential growth factor for maternal–embryo interactions due to its expression both in the female reproductive system and the embryo itself. However, due to its pleiotropic activity, it is extremely difficult to distinguish the effect of embryonic and maternal-derived protein on early embryo development. An indication that activin A derived from the reproductive tract of the female does contribute to embryo development comes from studies showing that exogenous activin A facilitates embryonic development in vitro. It was shown that treatment of cultured rodent, bovine, and goat embryos with recombinant activin A promotes embryo development, by releasing the “two-cell block,” increasing blastocyst cell number, reducing the time taken to reach the blastocyst stage, and improving hatching rates [18–22, 28]. In this work, we excluded the influence of exogenous activin A by investigating the requirement for the zygotic pool of this protein in the specification of primary cell lineages of the embryos cultured in vitro from the zygote to the blastocyst stage.

Using knockout mouse lines, we revealed that embryos deficient for functional zygotic activin A cultured in vitro (without the influence of exogenous activin A) developed successfully to the blastocyst stage. Moreover, the correct progression of development was also independent of exogenous protein, which is normally provided by maternal tissues, and embryo culture conditions (atmospheric or reduced oxygen level). These results led us to conclude that zygotic activin A is dispensable for the preimplantation development of the mouse embryo. However, it is known that before zygotic genome activation, embryonic development is dependent on maternally inherited RNAs and proteins [29, 30]. Since activin A is expressed both maternally and zygotically, we cannot exclude that activin A of maternal origin (originating from the oocyte) can rescue the zygotic deficiency or delay the development of the mutant phenotype in zygotic knockout embryos [31, 32]. Thus, the contribution of both maternal and zygotic gene expression to early embryo development during the preimplantation period requires further investigation.

Our results also indicate that although development to the blastocyst stage occurs without disruption, the implantation competence of activin A–deficient blastocysts may be compromised. So far, the role of activin A in the implantation process has been widely explored, but in the context of decidual rather than embryonic protein. It was shown that decidua-derived activin A promotes trophoblast cell outgrowth, differentiation, and invasiveness [4, 33]. Autocrine and paracrine activity of decidual activin A stimulates the production of cytokines and matrix metalloproteinases, facilitating blastocyst attachment and trophoblast penetration [34]. Moreover, activin A regulates the adhesive properties of trophoblast cells by modulating the expression of E- and N-cadherin [35, 36] and integrins [37]. Since the implantation is a strictly regulated process requiring crosstalk and synchrony between a receptive endometrium and an embryo, we focused on blastocyst competence for this process. Our data indicate that in the absence of zygotic activin A, the functionality of the TE can be disrupted as evidenced by the blastocyst outgrowth formation assay. Reduced invasion of trophoblast cells suggests that *Inhba*^KO/KO^ blastocysts can display impaired potential for implantation. During the initial stages of the implantation process, feto-maternal dialogue is mediated by the cell adhesion molecules, present on both the endometrial and trophoblast surfaces. Thus, we speculated that activin A plays a role during the early stages of implantation by affecting the adhesive properties of trophoblast cells. However, we failed to confirm this by immunostaining one of the adhesive proteins—integrin α2. Nevertheless, despite the potential importance of zygotic activin A in the implantation process, which indicated outgrowth assay in our study, activin A–deficient embryos undergo implantation and develop successfully to term [12]. It seems probable that maternal decidua is a main source of activin A for developing embryos and the presence of exogenously provided protein may explain the lack of severe developmental defects in the absence of activin A of embryonic origin. This hypothesis is supported by the results of our “rescue” experiment, as the difference between mutants and wild-type outgrowths was abolished by the presence of exogenous protein in the medium. Remarkably, this experiment also revealed that, despite the absence of activin A, the high dose of external protein could have a detrimental effect on the spreading of the outgrowth. An observation that the total surface area of *Inhba*^WT/WT^ and *Inhba*^KO/WT^ outgrowths cultured with activin A was reduced in comparison to their untreated counterparts suggests that precisely defined levels of this protein are needed for trophoblast invasion to occur correctly.

As we revealed that activin A deficiency may affect the functionality of the TE, our next step was to examine the competence of the EPI. We decided to check whether *Inhba*^KO/KO^ blastocysts can give rise to embryonic stem cells. Mouse and human ESCs have been shown to utilize different signaling pathways to maintain pluripotency. In contrast to hESCs, which show features of a developmentally later stage than the naïve pluripotency state and require exogenous activin A (added to the medium) for derivation and maintenance of the pluripotency [16, 17, 38, 39], data on the effect of exogenous activin A on the efficiency of obtaining mESCs lines are ambiguous. Activin A alone is unable to sustain ESCs in an undifferentiated state. However, supplementation of the culture medium with exogenous activin A and bone morphogenetic protein 4 increases the efficiency of obtaining mESCs exhibiting so-called enhanced pluripotency. The ESCs derived under these culture conditions were able to create chimeras, and in the tetraploid complementation assay, they generated individuals composed exclusively of ESCs [40]. Moreover, activin A in combination with the ERK1/MAPK pathway inhibitor was shown to promote mESCs propagation with naïve pluripotency features [41]. On the other hand, it has been shown that exogenous activin A has a harmful effect on the epiblast development and derivation of mESCs [14]. In turn, the role of endogenous activin A is unclear in both mouse and human ESCs. Our experiments constitute the first attempt to verify the importance of both endogenous and exogenous activin A for mouse ESCs. We revealed that ESCs were successfully derived, expanded, and maintained in an undifferentiated state in the absence of endogenous activin A regardless of the exogenous source of this protein. They were indistinguishable from wild-type ESCs in morphology and expression of lineage-specific markers, including pluripotency factors. A similar conclusion comes from studies in which mESCs were induced to overexpress SMAD6/7 or treated with an inhibitor of type I receptors for activin A. Such a treatment did not affect their pluripotency [42]. Since MEFs serving as a feeder layer for ESC culture also secrete activin A [38], we tested various culture conditions (*Inhba*^KO/KO^ vs *Inhba*^WT/WT^ MEFs) which allowed us to rule out that exogenous protein can compensate for the endogenous protein deficiency, thereby allowing ESC derivation from *Inhba*^KO/KO^ embryos and maintaining their undifferentiated state.

We also showed that activin A is dispensable for ESC pluripotency, as *Inhba*^KO/KO^ ESCs undergo differentiation in vitro into ectoderm, mesoderm, and endoderm. EB formation is often used as a method for initiating spontaneous differentiation toward the three germ layers [43–45]. We revealed that ESCs lacking functional activin A can form embryoid bodies expressing selected markers of all three germ layers both at the mRNA and the protein levels. Since ESCs induced to differentiate were cultured in suspension, without a feeder layer, they can serve as an in vitro model of postimplantation embryo devoid of the influence of exogenous activin. The use of such a model allowed us to check whether endogenous and exogenous sources of this protein are necessary for differentiation processes. It is still unresolved whether activin A is involved in the gastrulation of the mouse embryo in vivo. Activin A is believed to act as a mesoderm-inducing factor during early amphibian development [46]. However, in the mouse postimplantation embryo (E6.0–9.5), during mesoderm formation, activin A is not expressed, but high levels of this protein have been detected in the maternal decidual tissue surrounding the embryo [9–11]. Such an expression pattern suggests that exogenous activin A reaches an embryo and induces mesoderm formation in a paracrine manner. Bearing in mind that the embryoid body can mimic peri-implantation embryo, we could use it to investigate the requirement of zygotic activin A for the initial stages of gastrulation. The ability of ESCs lacking activin A and without supplementation of exogenous activin A to multidirectional differentiation suggests that this growth factor is not required for the early stages of postimplantation development of the mouse embryo. However, it should be remembered that we detected early markers of differentiation into three germ layers. It cannot be ruled out that the lack of activin does not affect the early stages of differentiation, but could disturb later stages. To check this, embryonic bodies should be placed in a culture so that they can adhere to the substrate and create outgrowths. Additional supplementation of the medium for the culture of such outgrowths would allow for directing the ESCs differentiation process and verifying whether the lack of activin prevents obtaining the desired cell type.

Taken together, although mouse preimplantation embryos and embryonic stem cells exhibit the expression of activin A, its disruption has no impact on the progress and dynamics of early development and the derivation, maintenance, and differentiation of embryonic stem cells. However, this protein of embryonic origin seems to be involved in the implantation of the embryo into the uterus. Our study may thus help elucidate the genetic causes that lead to implantation failure and, as a consequence, pregnancy loss.

## Materials and methods

### Generation of *Inhba*-KO mouse lines

Knockout *Inhba* mouse lines were generated in the Genome Engineering Unit of the International Institute of Molecular and Cell Biology (Warsaw, Poland) using the CRISPR/Cas9 method. The *Inhba* knockout mouse line (*Inhba*-KO) was established by a loss-of-function indel mutation in coding exon 1 of *Inhba* gene (c.129_130ins22bp, p.P44X—insertion of 22 bp between position 129 and 130 of cDNA—transcripts ID ENSMUST00000042603.13; ENSMUST00000164993.1) in C57BL/6/Tar genetic background and C57BL/6/Tar x CBA/Tar mixed background. The insert cassette contains EcoRI enzyme digestion sequences and STOP codon, which cause premature translation arrest in each of the three possible ORF sequences and result in a frameshift mutation. The presence of the mutation was confirmed by Sanger sequencing in the founder mouse and, after crossing, in *Inhba*^KO/WT^ mouse, and visualized using Benchling online software.

*Inhba*-KO mice were bred in the Animal Facility of the Faculty of Biology, University of Warsaw. All animal experiments were approved by the Local Ethics Committee for Experimentation on Animals no. 1, Warsaw, Poland (permission no 1336/2022) and were conducted under the ARRIVE guidelines and national regulations.

### Mouse genotyping

Mice were genotyped using the HotShot method [47] with minor modifications. For DNA isolation from ear punches, the volumes of alkaline and neutralization solutions were scaled up to 120 μl. Lysis time was reduced to 25 min. DNA extract (1 μl) was added to 9.5 μl of PCR mix containing Phusion HS II polymerase, Green HF buffer (ThermoFisher Scientific), 200 μM dNTPs, nuclease-free water, and 0.5 μM primers. For *Inhba*-KO mice forward (5′-CACAAACCTACAGCACTGAC-3′) and reverse (5′-CCACTTTACCCACATGAAGC-3′) primers were used and the amplification proceeded under the conditions: initial denaturation at 98°C for 3 min, followed by 35 repeated cycles with 98°C for 13 s, 63°C for 17 s, and 72°C for 9 s, and final extension at 72°C for 5 min. *Inhba*-KO mouse PCR products were digested with EcoRI enzyme at 37°C for 12 min, followed by electrophoresis separation in a 1.5% agarose gel. The wild-type allele results in a 482-bp product, while the *Inhba*-KO allele results in a 504-bp product and after digestion gives two bands of 332 and 171 bp.

### Isolation of embryos

To obtain zygotes, females carrying a mutation in one allele of the *Inhba* gene (*Inhba*^KO/WT^) were induced to superovulation with 10 IU of PMSG (pregnant mare’s serum gonadotropin; Intervet) followed after 48 h with 10 IU of hCG (human chorionic gonadotropin; Intervet) and were mated with *Inhba*^KO/WT^ males in the same genetic background (C57BL/6/Tar or C57BL/6/Tar x CBA/Tar mixed background). Females, in which vaginal plugs were detected, were autopsied 24 h after the hCG injection. Zygotes were recovered from the ampullae of oviducts and cleared of follicular cells using hyaluronidase (300 μg/ml; Sigma-Aldrich).

Blastocysts for ESC derivation were isolated after natural mating of C57BL/6/Tar x CBA/Tar *Inhba*^KO/WT^ females with males of the same genotype. Females, in which vaginal plugs were detected, were autopsied on E3.5, and their dissected uteri were flushed using an M2 medium with BSA (bovine serum albumin; Sigma-Aldrich).

### MEFs derivation and culture

To obtain *Inhba*-KO MEFs, E13 fetuses were isolated from C57BL/6/Tar x CBA/Tar *Inhba*^KO/WT^ females after natural mating with *Inhba*^KO/WT^ males. The uterus, placenta, and fetal membranes were dissected to release the fetuses. After head decapitation, the fragment of the tail was retained for genotyping. Subsequently, the visceral tissues were discarded and the remaining corpora were minced and digested with 0.25% trypsin–EDTA for 30 min at 37°C. The enzymatic activity was neutralized by adding MEF medium containing high-glucose Dulbecco modified eagle medium (DMEM), 10% fetal bovine serum (FBS), and 1% penicillin and streptomycin, and the tissue was pipetted up and down to get a single cell suspension. The cells were cultured in T-75 flasks until confluence and then frozen. They were then verified for the lack of activin A in the ELISA according to the manufacturer’s protocol.

### Measurement of activin A by ELISA

In brief, in-house-derived MEFs (*Inhba*^WT/WT^ and *Inhba*^KO/KO^) were seeded at 4 × 10^5^ cells in a 60-mm dish to near confluence. After 48 h of incubation, cells were washed once and the medium was changed to 4 ml of fresh serum-free medium for the next 24 h. The supernatant of each culture medium was collected and centrifuged at 1500 rpm (10 min, 4°C) to remove any debris. The concentration of mouse activin A in the culture medium of MEF cell lines was carried out by using Activin A Immunoassay (human/mouse/rat activin A Quantikine ELISA kit; R&D Systems, #DAC00B) according to the manufacturer’s instructions.

### ESC derivation and culture

Blastocysts flushed from the uteri with an M2 medium were transferred to single wells of 96-well culture dishes covered with gelatin (0.2% w/v; Sigma-Aldrich) and a feeder layer of growth-arrested wild-type or *Inhba*^KO/KO^ MEFs (inactivated by treatment with mitomycin C at 10 μg/ml for 2 h). The medium for ESC derivation was composed of KnockOut DMEM (Gibco) supplemented with 15% serum replacement (SR; Gibco) with the addition of nonessential amino acids (0.1 mM; Gibco), glutaMax (2 mM; Gibco), β-mercaptoethanol (0.1 mM; Sigma-Aldrich), penicillin (50 IU/ml), streptomycin (50 μg/ml), murine leukemia inhibitory factor (LIF, 1000 IU/ml; ESGRO, Chemicon International), PD9325901 (1 μM; Stemgent), CHIR99021 (3 μM; Stemgent), and FGFRi (2 μM). After 3–4 days of culture, blastocysts that formed outgrowths were disaggregated enzymatically and mechanically by incubation with 0.25% trypsin/EDTA (Gibco) for 5 min and subsequent pipetting. Resulting cell suspensions were transferred onto inactivated MEFs (wild-type or *Inhba*^KO/KO^) and were inspected daily for the appearance of primary colonies. Cultures that contained ESCs were expanded, processed for genotyping and karyotyping, and then frozen for further investigation.

### Formation of embryoid bodies

ESCs were separated from MEFs by the pre-plating procedure. To this end, cultures were washed twice in phosphate-buffered saline (PBS) and incubated in 0.25% trypsin/EDTA for 5 min. Then cells were suspended in a culture medium, plated again onto a culture dish covered with 0.2% gelatin, and then incubated at 37°C for 20–30 min, which allowed selective removal of fast attaching MEFs. If needed, pre-plating was repeated. The ESC-containing medium was used for EBs generation. EBs were generated using the hanging drop technique. In brief, 800 ESCs were suspended in 30-μl drops of ESC medium that lacked LIF. Drops were placed onto covers of culture dishes filled with PBS and cultured at 37°C. On day 2 of culture in hanging drops, EBs were transferred to low adhesive dishes (Medlab) allowing for their culture in suspension. At day 7 of culture, EBs were collected and fixed for immunofluorescence analysis or subjected to mRNA transcript analysis.

### Time-lapse imaging and morphokinetic analysis

The preimplantation development of *Inhba*-KO zygotes was subjected to time-lapse recording (every 10 min) under the PrimoVision imaging system (Vitrolife) enclosed in a standard embryo culture incubator maintaining constant culture conditions (37.5°C, 5% CO_2_). The embryos were cultured in 16-well dishes (Vitrolife) with 30-μl droplets of M16 medium (Sigma-Aldrich) for 4 days. Acquired images were analyzed with ImageJ software for morphokinetic parameters, which included (1) t_NEBD_—the time between the hCG injection and the nuclear envelope breakdown of pronuclei; (2) t_2_ to t_8_—periods between the hCG injection and the moment when the embryo reaches a certain number of cells; (3) t_comp_ and t_cavit_—the time between the hCG injection and beginning of compaction and cavitation, respectively; (4) m_1_—duration of the first embryonic cycle, i.e. the period between the disappearance of pronuclei and 2-cell stage; (5) cc_2_ and cc_3_—duration of the cell cycle for 2- or 4-cell stage blastomeres, respectively; (6) s_2_ and s_3_—the synchronicity of second and third rounds of cleavage divisions, respectively; and (7) s_M_—a period between compaction and cavitation when embryo remains in morula stage. To preserve the true distribution of genotypes for the isolated embryos, zygotes that did not fit into the dish for recording were cultured outside the imaging area and then processed as recorded embryos. Once filming was completed, all the embryos were individually fixed while preserving their identities. Subsequently, the embryos that reached the blastocyst stage were stained for the presence of cell lineage markers and after analysis under a confocal microscope, all blastocysts were individually genotyped. Embryos that were arrested in development were only fixed and genotyped. The data were collected from at least seven independent experiments for *Inhba*-KO embryos in C57BL/6/Tar x CBA/Tar and C57BL/6/Tar genetic background.

### Culture of embryos in hypoxic conditions

After isolation, the *Inhba*-KO C57BL/6/Tar x CBA/Tar zygotes were cultured in the EmbryoMax Advanced KSOM Embryo Medium (Sigma-Aldrich; MR-101-D) with the additional supplementation of D-(+)-glucose (Sigma-Aldrich), and essential amino acids without L-glutamine (EAAs; 50x; Gibco) to final 3.4 mM and 0.5× concentration, respectively. Embryos were cultured in a HypoxyLab incubator (Oxford Optronix) at 5% oxygen, maintained by purging the incubator with 90% N_2_, 5% CO_2_, and 5% O_2_. Embryos remained undisturbed in the incubator for 96 h, to the blastocyst stage, and then were immunostained for cell lineage markers.

### Blastocyst outgrowths formation and measurement of their area

For blastocyst outgrowth formation assays, C57BL/6/Tar x CBA/Tar blastocysts were cultured individually in ESC medium without LIF in 96-well cell culture plates (ThermoFisher Scientific) pre-coated with 0.2% gelatin (Sigma-Aldrich). The standard ESC medium contained knockout DMEM (ThermoFisher Scientific) supplemented with 15% FBS (ThermoFisher Scientific), streptomycin (50 μg/ml; ThermoFisher Scientific), penicillin (50 IU/ml; ThermoFisher Scientific), nonessential amino acids (0.1 mM; ThermoFisher Scientific), glutaMax (2 mM; Gibco), and β-mercaptoethanol (0.1 mM, Sigma-Aldrich). For the rescue experiments, blastocysts were cultured in ESC medium with 500 ng/ml recombinant activin A protein (R&D Systems, 338-AC-010). To maintain a constant concentration of activin A throughout the culture, the old ESC + activin A medium was gently drawn off and replaced with a fresh one on the third day of outgrowth culture. After 96 h of culture, the obtained outgrowths were photographed under a stereoscope microscope (Nikon) at 5× magnification. The proportion of adhered blastocysts and the total and ICM outgrowth area were examined to estimate the implantation capacity of blastocysts in vitro. Briefly, once the perimeter of the trophoblast or ICM cells was respectively selected using a computer mouse, the total outgrowth area and ICM area were calculated in px^2^ using ImageJ software, and converted to square micrometers using the conversion factor 1 px^2^ = 0.255 μm^2^, determined using a calibration slide. After acquiring the images, outgrowths were subsequently fixed and genotyped. Blastocysts that failed to form outgrowths were also subjected to genotyping to determine the efficiency of outgrowth formation for a given genotype. The data were collected from six independent experiments.

To detect cell lineage markers in outgrowths, blastocysts were cultured as above in an 8-well chambered coverslip with polymer coverslip-bottom (Ibidi). At the end of 96 h of culture, the obtained outgrowths were prepared for confocal analysis according to the immunostaining protocol. At the end of the experiments, the outgrowths were individually genotyped.

### Immunofluorescent staining

Blastocysts, outgrowths, ESCs, and EBs were fixed in 4% paraformaldehyde (ThermoFisher Scientific) in Ca^2+^- and Mg^2+^-free PBS (Biomed) for 30 min, permeabilized with 0.5% Triton X-100 (Sigma-Aldrich) for 15–30 min, and placed in 10% FBS (ThermoFisher Scientific) or 3% BSA (Sigma-Aldrich) with 0.01% sodium azide (Honeywell Fluka) at 4°C. The following primary antibodies were used: mouse monoclonal antibody (1:50, BioGenex, MU392A-UC; RRID:AB_2923402) or rabbit monoclonal (1:200, Cell Signaling Technology, 12306S; RRID:AB_2797879) antibody against CDX2, rabbit polyclonal antibody against SOX2 (1:100, Abcam, ab97959; RRID:AB_2341193), rat monoclonal antibody against Nanog (1:200, ThermoFisher Scientific, 14-5761-80; RRID:AB_763613), goat polyclonal antibody against SOX17 (1:100, R&D Systems, AF1924; RRID:AB_355060), goat polyclonal antibody against GATA4 (1:100, R&D Systems, AF2606; RRID:AB_2232177), mouse monoclonal antibody against OCT4 (1:100, Santa Cruz Biotechnology, sc-5279; RRID:AB_628051), rat monoclonal antibody against TROMA1 (1:50, Developmental Studies Hybridoma Bank, AB_531826; RRID:AB_531826), goat polyclonal antibody against Brachyury (1:200, R&D Systems, AF2085; RRID:AB_2200235), and rabbit monoclonal antibody against integrin α2 (1:100, Abcam, ab181548; RRID:AB_2847852). After 24 h of incubation with primary antibodies at 4°C, the blastocysts, outgrowths, ESCs, and EBs were washed three times for 15 min each in Ca^2+^- and Mg^2+^-free PBS and then incubated for 1.5 h at room temperature in a mix of matching secondary antibodies: donkey anti-mouse IgG conjugated with Alexa Fluor 594 (1:200, Invitrogen, A21203; RRID:AB_2535789), donkey anti-rabbit conjugated with Alexa Fluor 647 (1:200, Invitrogen, A31573; RRID:AB_2536183), donkey anti-goat IgG conjugated with Alexa Fluor 488 (1:200, Invitrogen, A11055; RRID:AB_2534102), donkey anti-rat IgG conjugated with Alexa Fluor 594 (1:200, Invitrogen, A-21209; RRID:AB_2535795), and goat anti-rat IgG conjugated with Alexa Fluor 633 (1:200, Invitrogen, A21094; RRID:AB_2535749). All primary and secondary antibodies were diluted in a blocking solution. After being rewashed in PBS, chromatin was stained with Hoechst 33342 (20 μg/ml in PBS, ThermoFisher Scientific), Chromomycin A3 (10 μg/ml, Sigma-Aldrich), or DRAQ5 (10 μM w PBS; Biostatus Ltd.) for 20 min at 37°C. The confocal analysis for blastocysts, ESCs, and embryoid bodies was performed on glass-bottom dishes (MatTek Corporation) and for outgrowths in chambered coverslip with polymer coverslip-bottom (Ibidi) in 10% FBS. Images were acquired with a Nikon Ti2-U microscope with focus motor assembly (Prior Scientific Instruments) using the rescan confocal microscopy module RCM1 (Confocal.nl) with an ORCA-Flash4.0 LT + camera (Hamamatsu) and iChrome CLE 50 laser engine (Toptica) under 10× and 25× with immersion objectives (Nikon). For each blastocyst and embryoid body, z-stacks were collected with 2.5-μm intervals between optical sections. The confocal images were analyzed using the ZEN 2.3 (blue edition) software and blastocyst cell counting was semiautomated with Imaris software.

### Genotyping of embryos, outgrowths, and ESC lines

At the end of each experiment, embryos, outgrowths, and ESC lines were genotyped according to the kit manufacturers’ instructions with minor modifications. Before DNA extraction from ESCs, the cells were suspended in PBS at a concentration of 1 million cells/100 μl. The 1 μl of suspensions (~10,000 ESCs) was used for DNA isolation, conducted as follows. For each sample, DNA was extracted using the Extract-N-Amp Tissue PCR Kit (Sigma-Aldrich) in a 5.5-μl mixture of Extraction and Tissue Preparation Solutions (4:1). Lysis was performed at 56°C for 30 min, 24°C for 5 min, and 95°C for 5 min, then 4.4 μl of N-Solution was added to each sample to stop the reaction. PCR was conducted using the Fast Cycling PCR Kit (Qiagen). Crude DNA extract was added to the PCR reaction mix containing the manufacturer’s PCR Mix, Q solution, nuclease-free water, and 0.5 μM primers. The amplification proceeded under the following conditions: initial denaturation at 95°C for 4 min, followed by 46 repeated cycles at 95°C for 30 s, 58°C for 45 s, and 68°C for 45 s, and final extension at 72°C for 10 min. For PCR of *Inhba*-KO embryos, outgrowths, ESCs, and MEFs, forward (5′-CTTTGGCTGAGAGGATTTCTG-3′) and reverse (5′-CCACTTTACCCACATGAAGC-3′) primers were used. Then, PCR products were digested with EcoRI enzyme (ThermoFisher Scientific) at 37°C for 20 min, followed by electrophoresis separation in a 2% agarose gel. The wild-type allele results in a 277-bp product, while the *Inhba*-KO allele results in a 299-bp product and gives two bands of 171 and 128 bp after digestion with EcoRI.

### RT-qPCR from embryoid bodies

For gene expression analysis in the embryoid bodies, total RNA was extracted and purified using the RNAqueous-Micro Kit (Ambion) according to the manufacturer’s instruction with modification of reagents volumes. The 50 embryoid bodies in 100 μl of lysis buffer were used for one sample. The volume of 100% ethanol was scaled to 125 μl, and a total volume of 20 μl solution was used for the elution of RNA from the filter. The reverse transcription was preceded by Oligo(dT)12–18 (ThermoFisher Scientific) primers’ hybridization to RNA at 70°C for 10 min in a T100 thermocycler (Bio-Rad). The synthesis of cDNA was performed using 200 U of Superscript II reverse transcriptase (Invitrogen), 40 U of RNA-ase inhibitor, 0.5 mM dNTPs, RT buffer, and 0.1 M Dithiothreitol (DTT; Invitrogen). Reactions were carried out in 20 μl in a thermocycler according to the program: 42°C for 50 min, 70°C for 15 min. The obtained cDNA was diluted twofold with nuclease-free water. Quantitative PCR was performed using specific Taqman probes (ThermoFisher Scientific) for *Pax1* (Mm_00435490_m1), *Pax6* (Mm00443081_m1), *Zic1* (Mm00656094_m1), *Eomes* (Mm01351984_s1), *Foxa2* (Mm01976556_s1), *Mixl1* (Mm00489085_m1), *Msgn1* (Mm00490407_s1), *Mesp2* (Mm00655937_s1), and *Nodal* (Mm00443040_m1) genes, in the presence of TaqMan Gene Expression Master Mix (ThermoFisher Scientific) and nuclease-free water. The reaction was conducted in a StepOne Real-Time PCR System thermocycler (Life Technologies), in duplicate for each gene, according to the program: 50°C for 2 min, 95°C for 10 min, followed by 50 repeated cycles with 95°C for 15 s and 60°C for 1 min. Data were analyzed using the 2^−ΔCT^ calculation method [48] and normalized to *β-actin* mRNA level (Taqman Mm01205647_g1). The experiment was repeated three times.

### Statistical analysis

Statistical analysis was conducted using R Statistical Software (v4.0.0; R Core Team, 2021). All quantitative data were verified for normal distribution by the Shapiro–Wilk test, while the homogeneity of variances was assessed using Levene test for multiple groups or Fisher test (test *F*) for two group experiments. Depending on the results of the above tests, a two-tailed Student *t*-test for independent samples or a Mann–Whitney *U*-test was used to analyze significant differences between the two groups. The comparison among multiple groups was performed by one-way ANOVA test or Kruskal–Wallis test when the assumptions for the parametric test were not met. Turkey test and Dunn test were used as post hoc tests, respectively. The comparison among qualitative data, e.g. efficiency of blastocyst and outgrowth formation between different genotypes and to expected distribution of genotypes, was performed by the Chi-square test and Chi-square goodness-of-fit test, respectively. In all tests, a value of *P* = 0.05 was considered a significant threshold.

## Supplementary Material

Winek_et_al_Supplementary_information_revision_ioae156

Movie1_ioae156

Movie2_ioae156

## Data Availability

The data underlying this article are available in Dane Badawcze UW Repository, at https://doi.org/10.58132/Z8RQD1.
