# Supplementary material for: Zygotic activin A is dispensable for the mouse preimplantation embryo development and for the derivation and pluripotency of embryonic stem cells
Source: Biol Reprod. 2024 Nov 6;112(1):31–45. doi: 10.1093/biolre/ioae156 (PMC11736436; doi:10.1093/biolre/ioae156)
Supplement: Winek_et_al_Supplementary_information_revision_ioae156 [file winek_et_al_supplementary_information_revision_ioae156.pdf]

Supplementary information

Zygotic activin A is dispensable for the mouse preimplantation embryo development and for the derivation and pluripotency of embryonic stem cells†

*Eliza Winek[1], Lidia Wolińska-Nizioł[1,3], Katarzyna Szczepańska[1,3], Anna Szpakowska[1], Olga Gewartowska[2], Izabela Wysocka[1], Magdalena Grzesiak[1], Aneta Suwińska[1]**

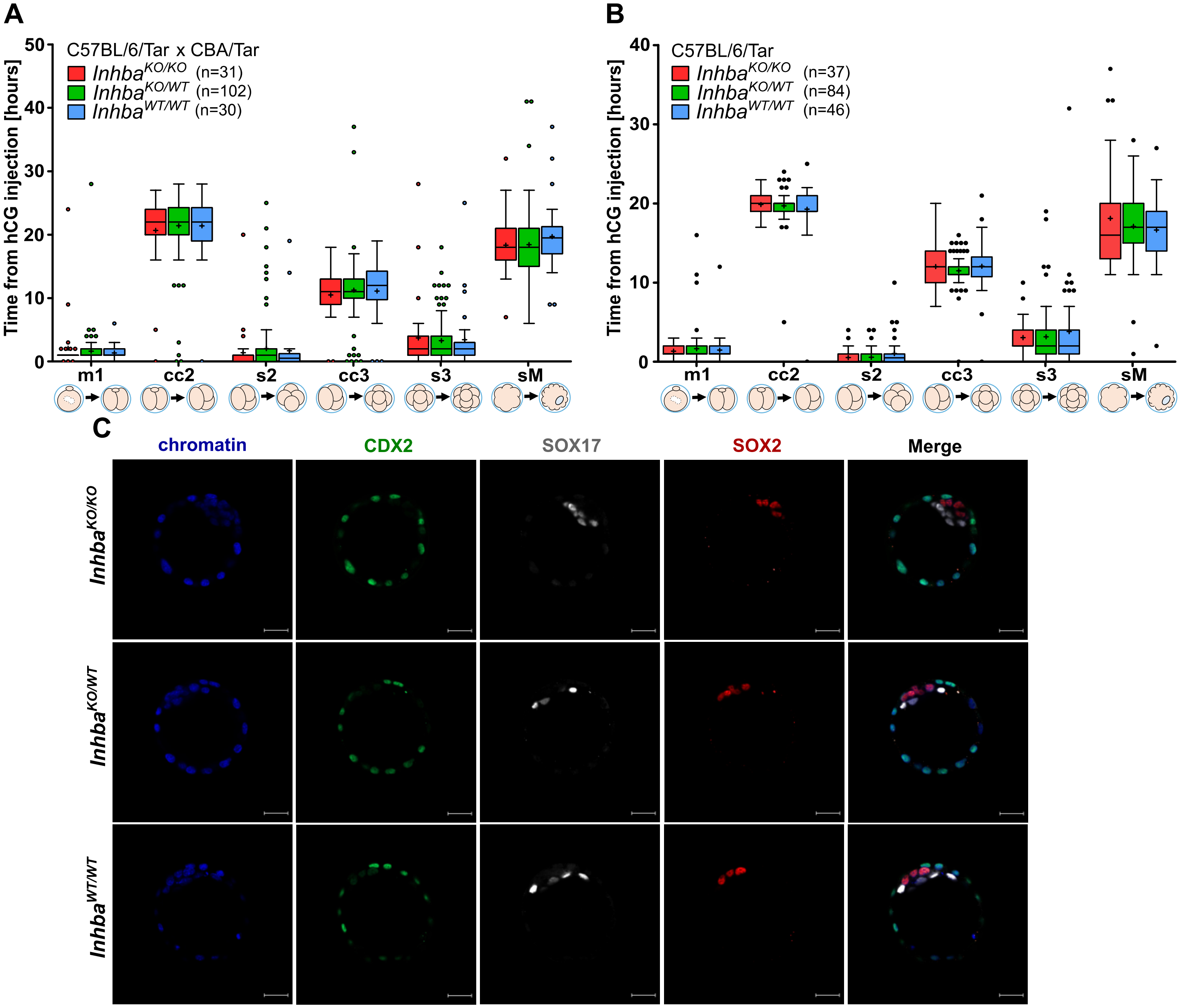

Fig. S1. Embryos deprived of zygotic and exogenous activin A have a comparable course of preimplantation development to control embryos. Related to Fig. 2. (A and B) Comparison of supplementary morphokinetic parameters between *Inhba*$^{KO/KO}$ and control – *Inhba*$^{KO/WT}$ and *Inhba*$^{WT/WT}$ embryos of the *Inhba*-KO mouse line in (A) F1(C57BL/6/Tar x CBA/Tar) and (B) C57BL/6/Tar genetic background. The value of individual parameters was calculated as the number of hours between: m1 – the disappearance of pronuclei and 2-cell stage i.e., duration of the first embryonic M-phase; cc2 and cc3 – the time between 2-cell and 3-cell stage, and between 3-cell stage and 5-cell stage, i.e. duration of the second and third cell cycle, respectively; s2 and s3 – the cleavage timing between progeny in the different cell generations i.e. synchronicity of second and third rounds of cleavage divisions, respectively; sM

– compaction and cavitation, the period during which the embryo remains in the morula stage. On the boxplots, the middle lines represent medians, the cross shows the mean value, the hinges indicate the interquartile range, the whiskers represent the minimum and maximum values in the group and the dots show outliers. $p>0.05$ reported for comparison in Kruskal-Wallis test (C) Confocal images of blastocysts in C57BL/6/Tar genetic background immunostained with antibodies against CDX2 (trophectoderm), SOX17 (primitive endoderm), SOX2 (epiblast), and chromatin stained with Hoechst 33342. Scale bar: 20 µm.

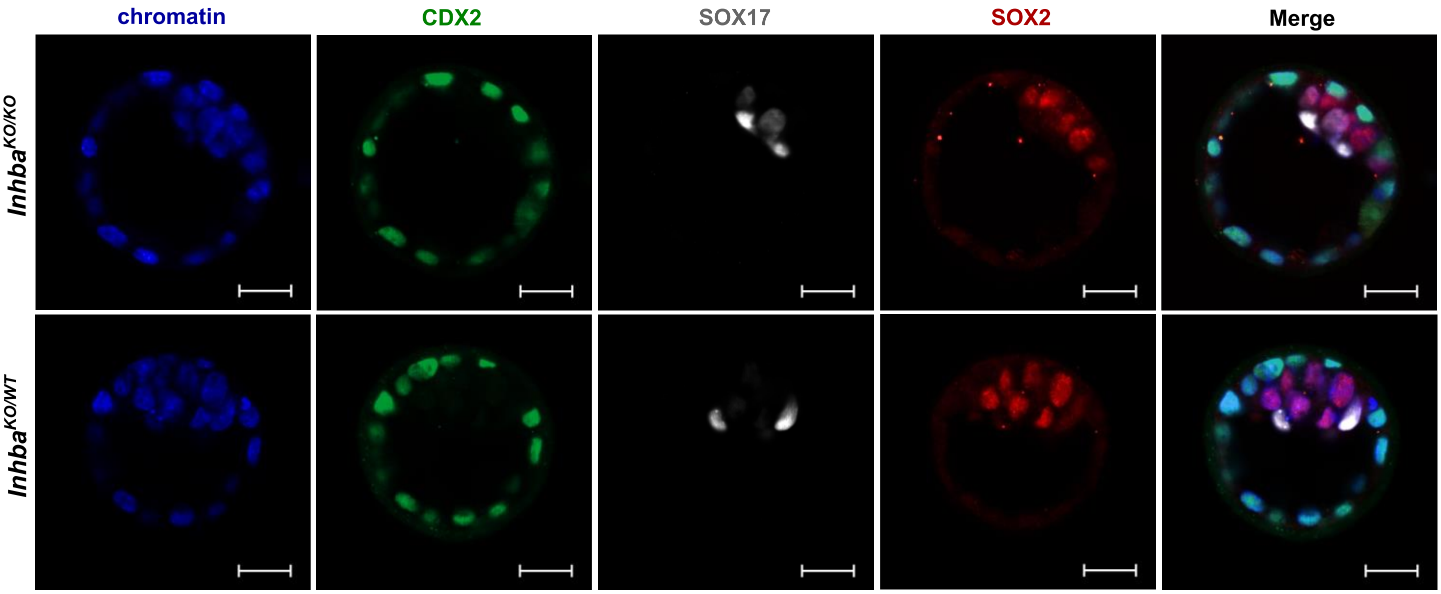

Fig. S2. Embryos deprived of zygotic and exogenous activin A form a blastocyst when culture conditions mimic those in vivo. Confocal images of *Inhba*$^{KO/KO}$ and *Inhba*$^{KO/WT}$ embryos cultured in hypoxic conditions in KSOM medium enriched by glucose and EAAs. Blastocysts were immunostained with antibodies against trophectoderm marker CDX2, primitive endoderm marker SOX17, and epiblast marker SOX2, with chromatin stained with Hoechst 33342. Scale bar: 50 µm.

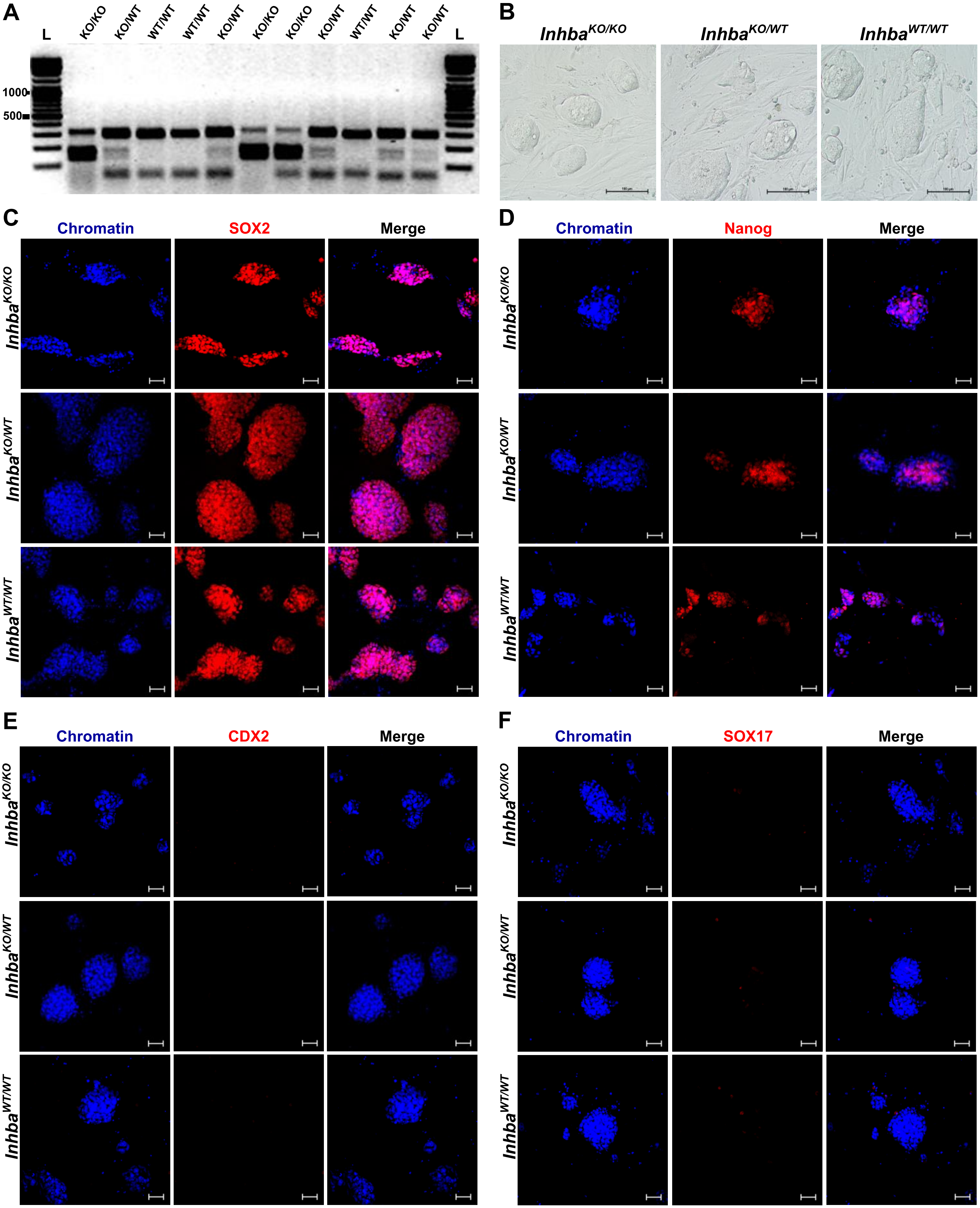

Fig. S3. Activin A-deficient ESCs can be derived and in the undifferentiated state upon the feeder layer of wild-type MEFs. (A) Gel image for genotyping derived *Inhba*$^{KO/KO}$, *Inhba*$^{KO/WT}$, and *Inhba*$^{WT/WT}$ ESCs of *Inhba*-KO line. The wild-type allele results in a 277 bp product, while the *Inhba*-KO allele results in a 299 bp product and gives two bands of 171 and 128 bp after digestion with

EcoRI. The higher band in *Inhba*$^{KO/KO}$ is the nondigested product for the *Inhba*-KO allele (299 bp). (B) Bright-field images of obtained ESC lines upon the wild-type MEFs. Scale bar: 100 µm. (C-F) Confocal images of ESCs immunostained with antibodies against epiblast markers (C) SOX2 and (D) Nanog; (E) trophectoderm marker CDX2 and (F) primitive endoderm marker SOX17, with chromatin stained with Chromomycin A3. Confocal images scale bar: 20 µm.

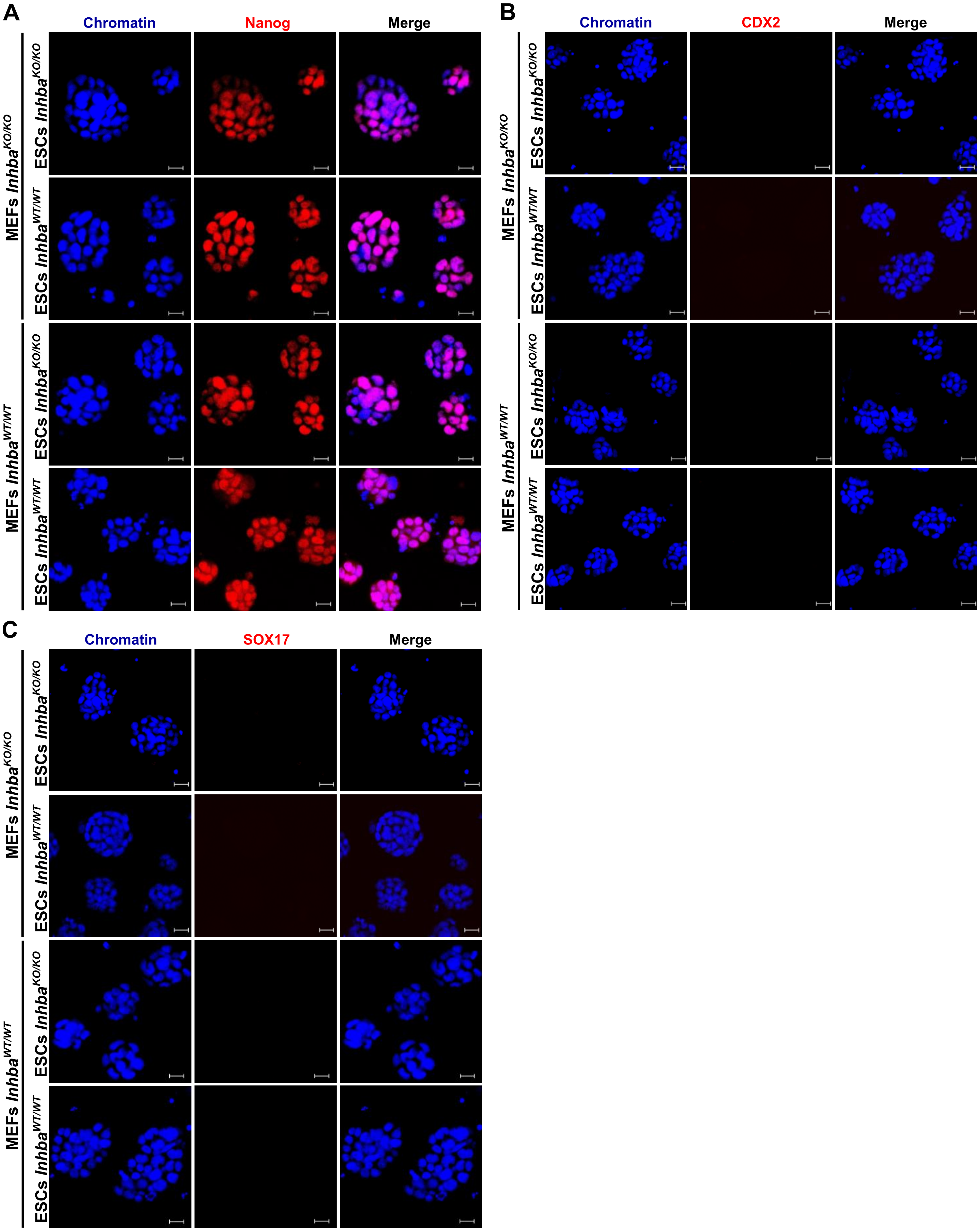

Fig. S4. Mouse ESCs can be established in the absence of endogenous activin A, and exogenous protein provided by the MEF feeder layer. Related to Fig. 4. Confocal images of ESCs immunostained with antibodies against (A) epiblast marker Nanog; (B)

trophectoderm marker CDX2 and (C) primitive endoderm marker SOX17, with chromatin stained with Chromomycin A3. Scale bar: 20 μm.
